# Supplementary material for: Photochemically responsive polymer films enable tunable gliding flights
Source: Nat Commun. 2024 Jun 1;15:4684. doi: 10.1038/s41467-024-49108-0 (PMC11144244; doi:10.1038/s41467-024-49108-0)
Supplement: Supplementary file 1 — Supplementary Information [file 41467_2024_49108_MOESM1_ESM.pdf]

Supplementary Information for

Photochemically responsive polymer films enable tunable gliding flights

Jianfeng Yang,[1] M. Ravi Shankar,[2] Hao Zeng[1]*

Affiliation:

[1]Light Robots, Faculty of Engineering and Natural Sciences, Tampere University, P.O. Box 541, FI-33101 Tampere, Finland.

[2]Department of Industrial Engineering, Swanson School of Engineering, University of Pittsburgh, Pittsburgh, PA 15261, USA.

*Correspondence to hao.zeng@tuni.fi

This PDF file includes:

Supplementary Figures. 1 to 24
Supplementary Table 1-2
Supplementary Methods
Supplementary References

Additional supplementary material for this manuscript includes:

Supplementary Movies 1-4

1. Supplementary Figures

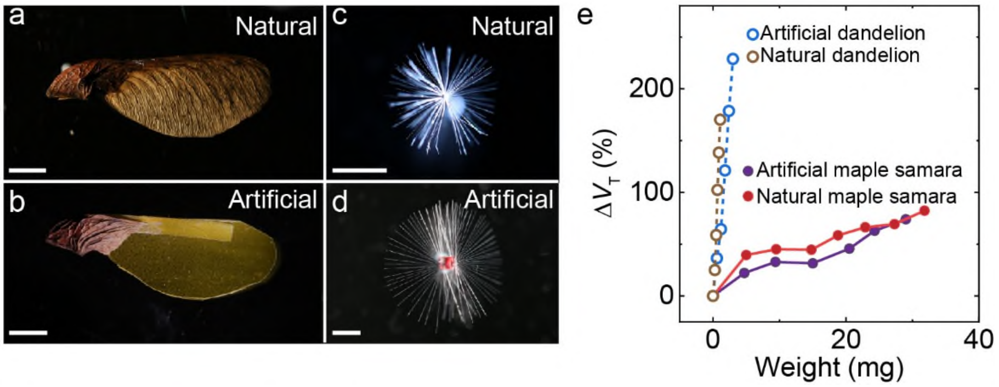

Supplementary Fig. 1 | Comparison of the load-carrying capacities. Photographs of natural maple samaras (**a**) and artificial seed (**b**). Photographs of natural (**c**) and artificial (**d**) dandelion seeds. (**e**) The changes of terminated velocity ΔV_T of natural maple samaras and artificial seeds, natural and artificial dandelion seeds along addition of weight. All the scale bars are 5 mm.

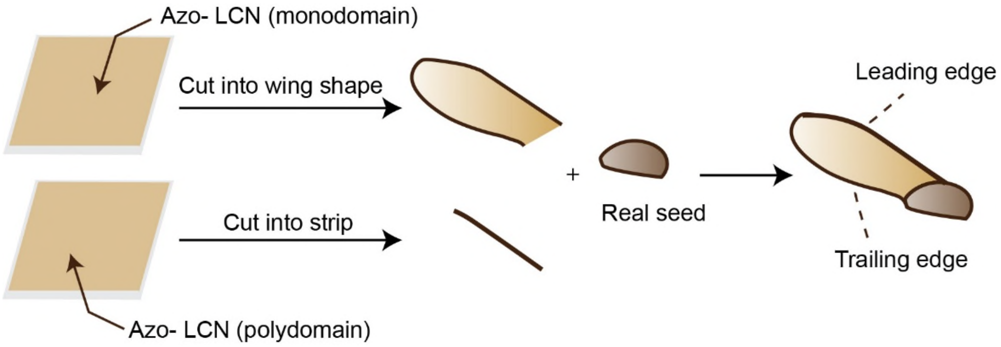

Supplementary Fig. 2 | Schematic drawing of steps of fabrication of artificial seed.

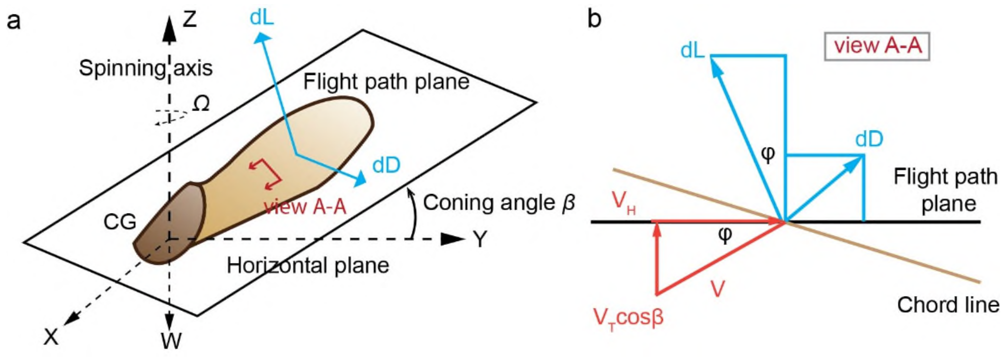

Supplementary Fig. 3 | Forces acting on the maple samara. The list of motion parameters: W, mass of maple samara; dL, sectional lift; dD, sectional drag; β, coning angle; Ω, spinning rate; CG, center of gravity, r, length of maple samara. In the persistent vertical flight of an autorotating seed, equilibrium is established between the vertical aerodynamic force component and the mass of the maple samara. The figure provides an overview of the forces and motion parameters involved in autorotation flight. Even during the vertical descent of an autorotating seed, the orientation of the samara blade plane is not perpendicular to the vertical axis of rotation. The tangent plane to the conical surface formed by the samara blade's trajectory (referred to as the flight path plane in this study) introduces a coning angle denoted as β with respect to the horizontal plane, as depicted in (**a**). The flight path plane forms an angle of $\tan^{-1}(V_T\cos\beta/r\Omega)$ with the resultant relative wind. This wind vector V results from the vector sum of the rotational velocity ($V_H = r\Omega$) and the descent velocity ($V_T\cos\beta$) components. This angle is labelled as φ in (**b**). The aerodynamic force is further dissected into lift force dL and drag force dD. The forces are balanced when:
$\int_{y=-Y_b}^{y=Y_t}(\mathrm{d}L\cos\varphi + \mathrm{d}D\sin\varphi)\cos\beta \mathrm{d}y - W = 0$ and $\int_{y=-Y_b}^{y=Y_t}(\mathrm{d}L\sin\varphi - \mathrm{d}D\cos\varphi)y\cos\beta \mathrm{d}y = 0$.
Here, the distance between the spinning axis of autorotation and the wing tip of the samara, represented as Y_t, while Y_b, denoting the distance between the spinning axis and the root tip of the samara. (**a**, **b**) Reproduced with permission.[1] Copyright 2022, Springer Nature. All rights reserved.

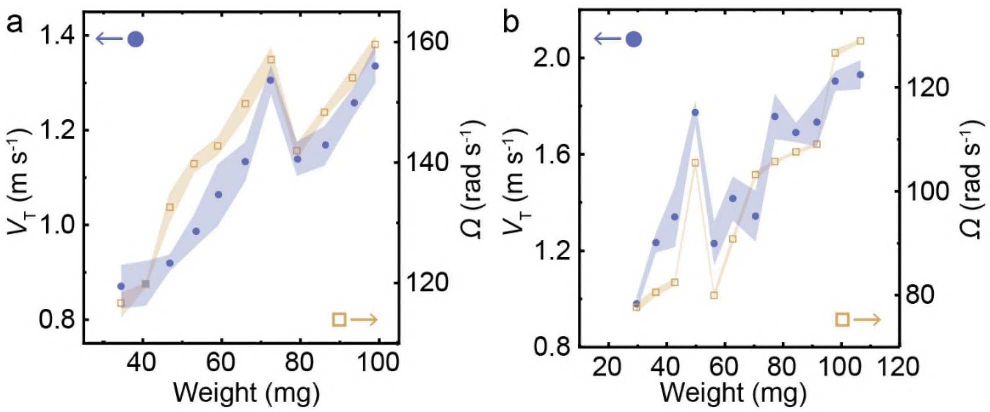

Supplementary Fig. 4 | The terminal velocities of natural maple samara and artificial seed upon different loadings. (**a**) The terminal velocity (V_T) and spinning rate (Ω) of a natural maple samara upon different loadings. (**b**) The V_T and Ω in an artificial seed upon different loadings. The mass is changed by adding weight onto the roots. The error bars are displayed as mean values +/- standard deviation (n = 3 independent experiments).

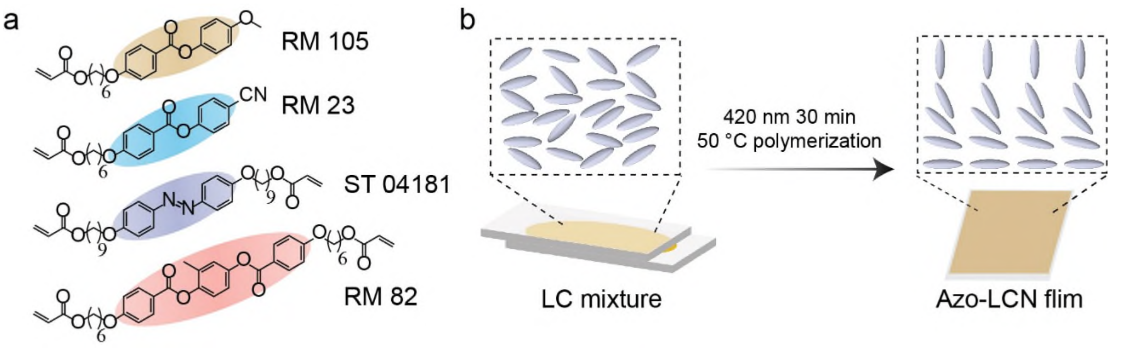

Supplementary Fig. 5 | Synthetic steps of azo-LCN. (**a**) Chemical structures of all molecules in use. Pre-cured mixture contains 52 mol% 4-Methoxybenzoic acid 4-(6-acryloyloxyhexyloxy) phenyl ester (RM 105), 18 mol% 4[4[6-Acryloxyhex-1-yl) oxyphenyl]carboxybenzonitrile (RM 23), 21 mol% diacrylate crosslinker 1,4-Bis-[4-(6-acryloyloxyhexyloxy)benzoyloxy]-2-methylbenzene (RM 82), 6 mol% 4,4'-Bis[9-(acryloyloxy)nonyloxy]azobenzene (ST 04181) and 1.5 mol% of photoinitiator. (**b**) Schematic drawing of the azo-LCN film preparation process and molecular alignment.

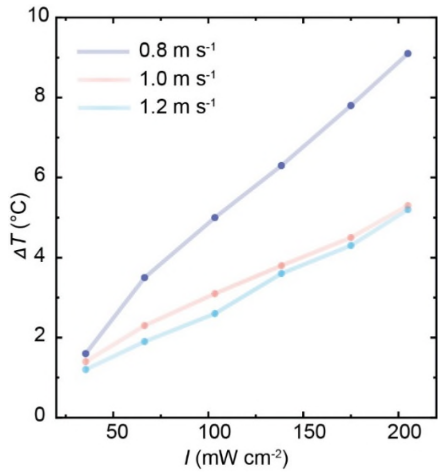

Supplementary Fig. 6 | The nearly isothermal condition upon photochemical actuation. The change of the temperature of azo-LCN film along UV irradiation intensity at different wind speeds.

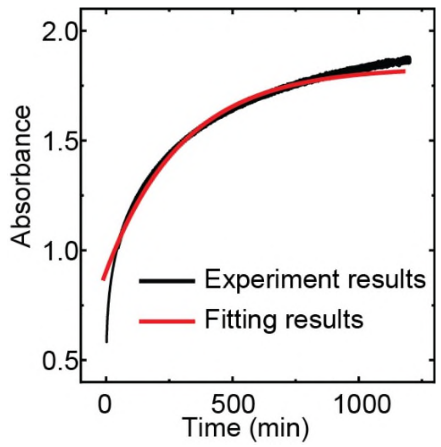

Supplementary Fig. 7 | The isomerization kinetics of azo-LCN film. Thermal relaxation (cis-to-trans) of the azobenzene absorption of azo-LCN film at room temperature, by monitoring absorbance at 360 nm.

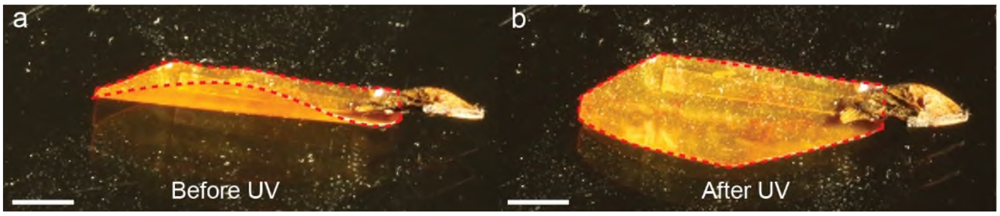

Supplementary Fig. 8 | The shape changes of the artificial seed wing. Top view photographs of artificial seed before (**a**) and after (**b**) UV illumination. UV: 240 mW cm^{-2}, 12 s The scale bars are 5 mm.

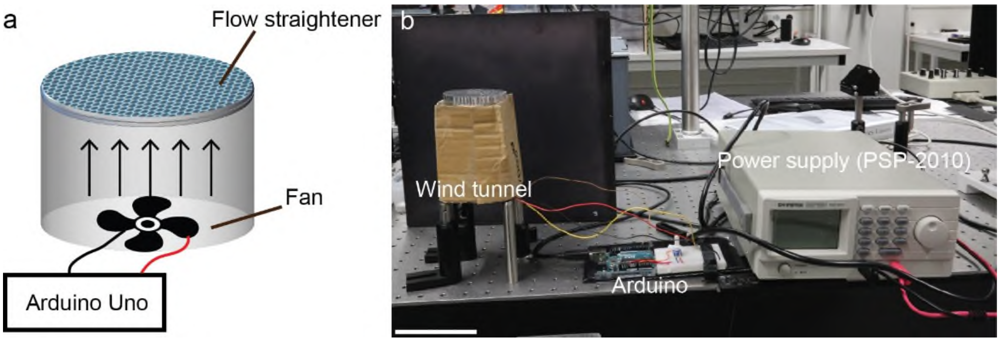

Supplementary Fig. 9 | The setup for wind tunnel experiment. (**a**) Schematic drawing of the setup of wind tunnel. (**b**) Photograph of experimental set up in laboratory. The scale is 10 cm.

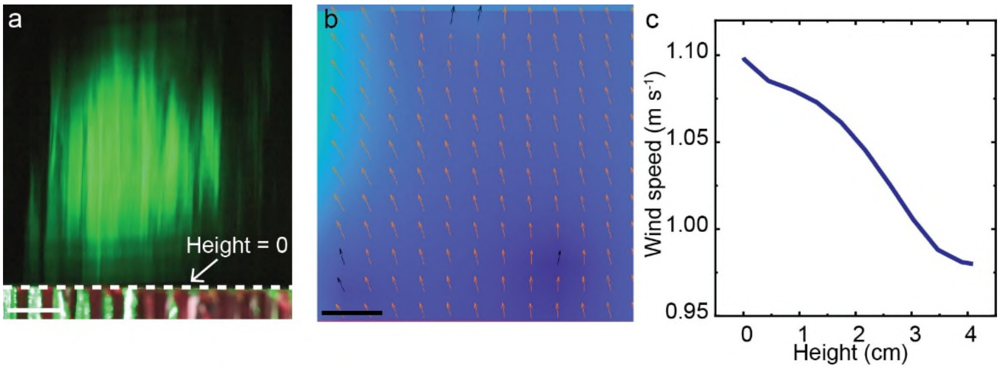

Supplementary Fig. 10 | The wind velocity gradient above the wind tunnel. (**a**) The photo of the wind flow at the wind tunnel output. The air is seeded with water droplets from humidity generator. The flow is visualized upon laser sheet illumination. (**b**) The particle image velocimetry test of wind flow. (**c**) The change of mean velocity of the wind along the height. All scale bars are 1 cm.

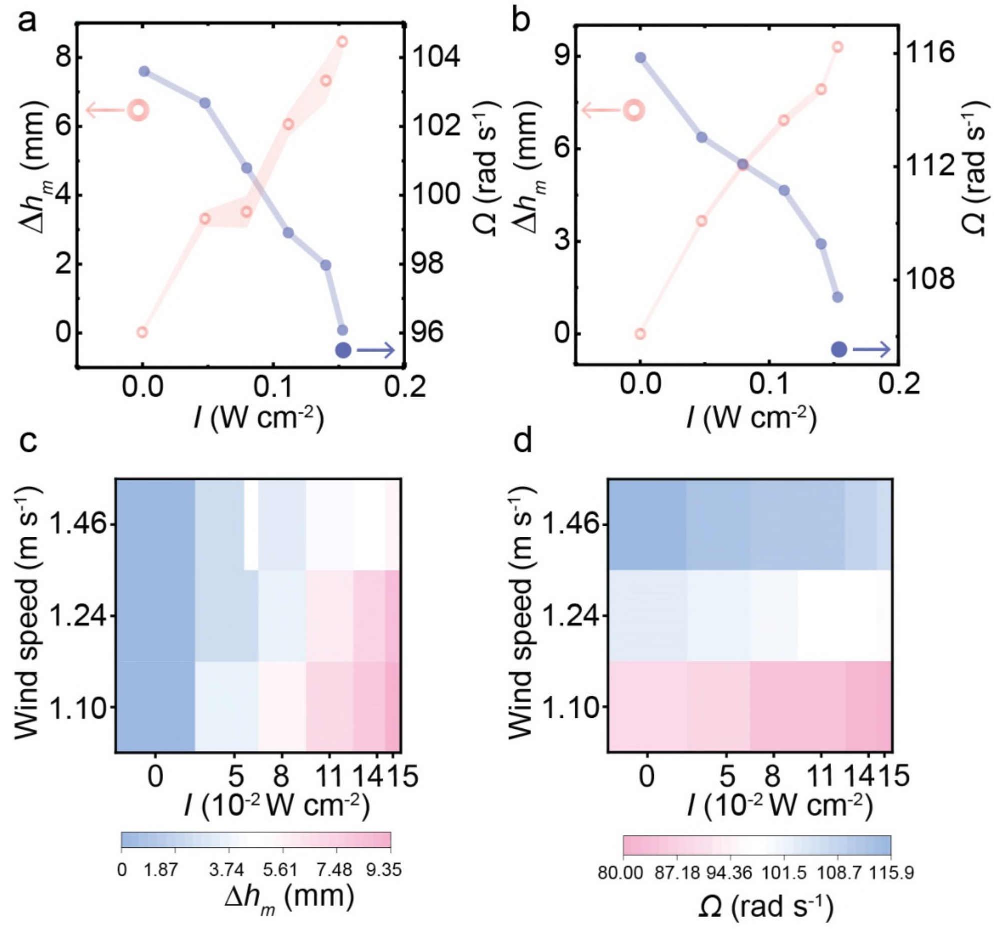

Supplementary Fig. 11 | The change of object height and spinning rate upon different wind flows and light intensities. The maximal change of the height Δh_m and spinning rate Ω of artificial seed upon different light intensities, at wind speed of 1.24 m s^{-1} (**a**) and 1.46 m s^{-1} (**b**). The error bars are displayed as mean values +/- standard deviation (n = 3 independent experiments). The color map summarizes the change in height (**c**) and spinning rate (**d**) of artificial seed at different wind speeds and UV light intensities.

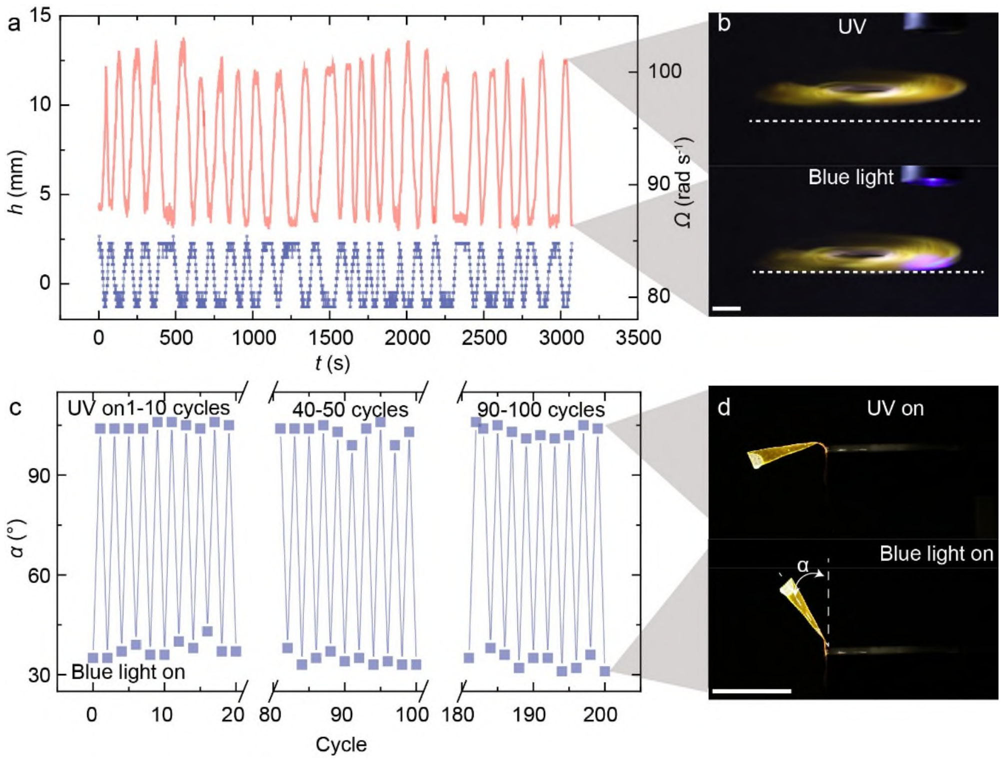

Supplementary Fig. 12 | The cycle test of artificial seed. (**a**) The change of *h* (red line) and *Ω* (blue dot) of artificial seed upon altering the UV and visible excitations in 25 cycles. UV: 360 nm, 150 mW cm^{-2}; visible light: 460 nm, 400 mW cm^{-2}. (**b**) The snapshot images of an artificial seed with reversible height change by altering the illumination. (**c**) Deformation of an azo-LCN strip during a hundred light actuation cycles. UV: 360 nm, 150 mW cm^{-2}; visible light: 460 nm, 400 mW cm^{-2}. (**d**) Photographs of light-induced deformation of an azo-LCN strip. All scale bars are 5 mm.

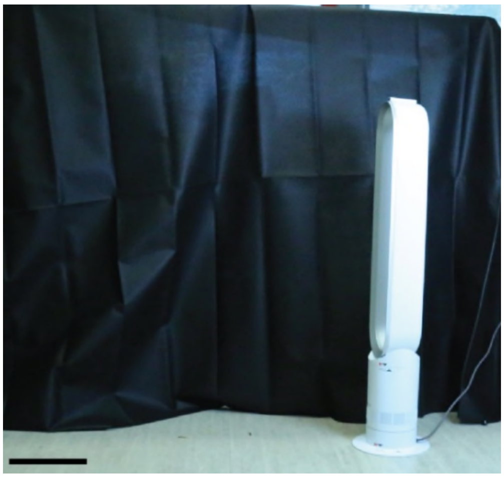

Supplementary Fig. 13 | The dispersal experiment with the help of a crosswind flow. Photograph of the free descent experiment of artificial seeds indoor. The scale bar is 20 cm.

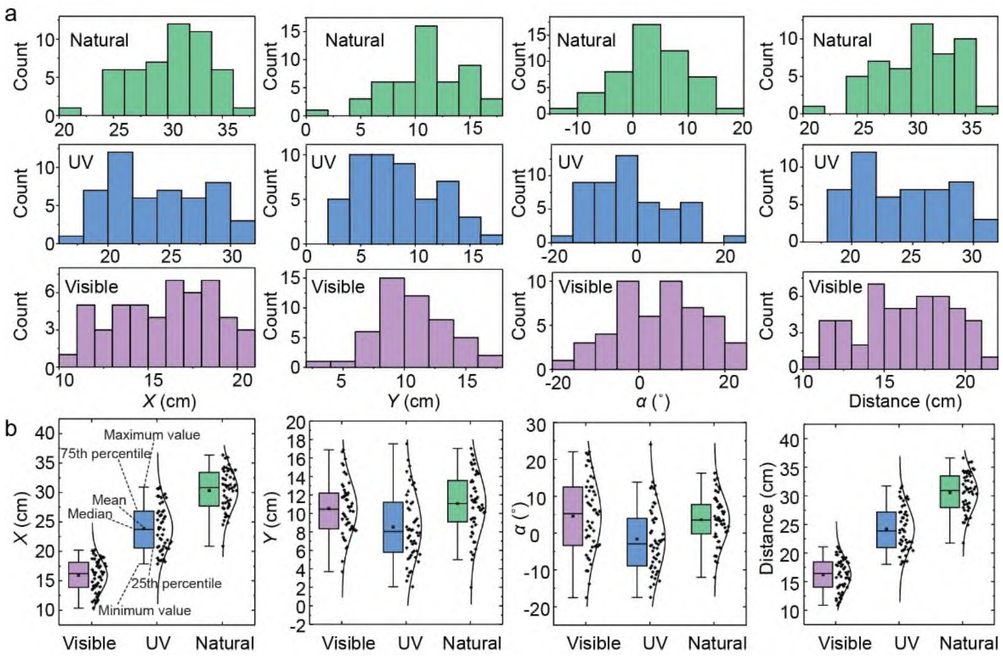

Supplementary Fig. 14 | The statistics of landing point distribution. The landing point is recorded on the substrate at X-Y plane, with horizontally traveling distance, *d*, and directional angle, α. (**a**) Counts of landing point in X- and Y- directions, α and *d*, upon UV and visible light irradiation. (**b**) Statistics of landing point in X- and Y- directions, α and *d*, for natural maple samara (green), artificial seed after UV irradiation (blue) and artificial seed after the visible light irradiation (purple).

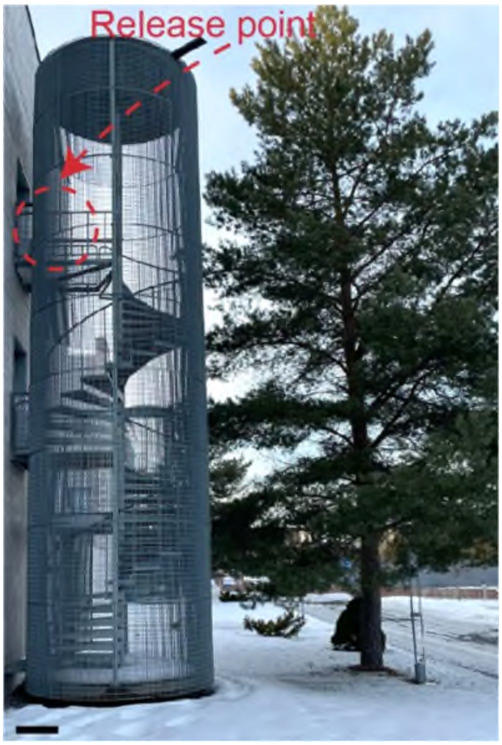

Supplementary Fig. 15 | The dispersal experiment in outdoor conditions. Photograph of the free-descent experiment of multiple artificial seeds in real-world environments. The releasing point is 14 meter above the ground. The scale bar is 1 m.

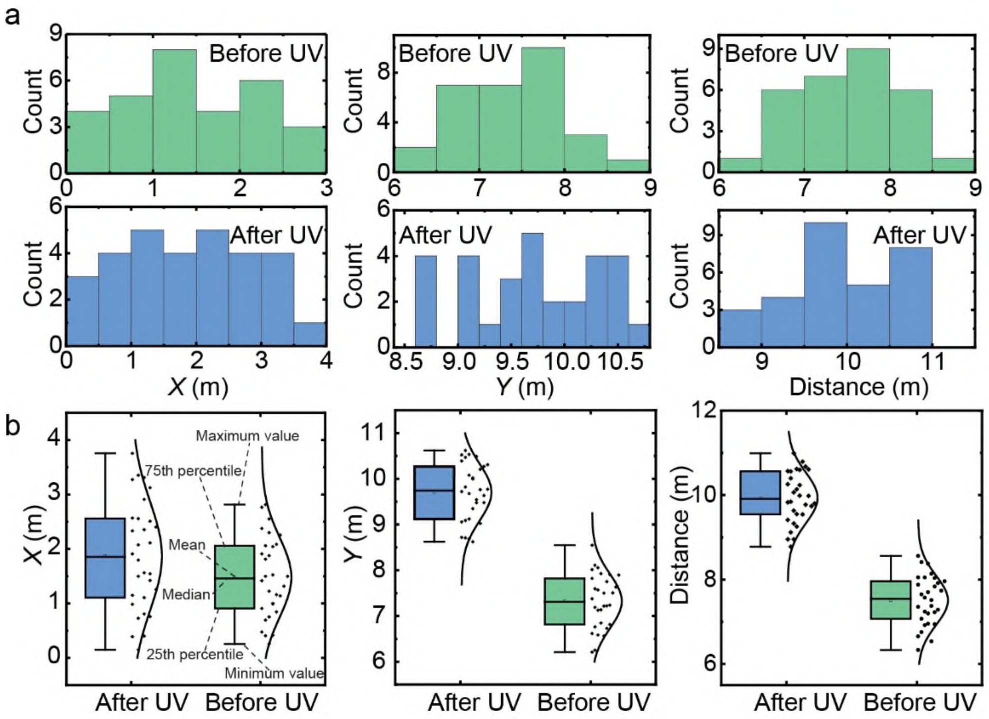

Supplementary Fig. 16 | The statistics of landing point distribution outdoor. The landing point is recorded on the substrate at X-Y plane, with horizontally traveling distance, *d*. (**a**) Counts of landing point in X- and Y- directions and *d* before UV and after UV irradiation. (**b**) Statistics of landing point in X- and Y- directions and *d*, for artificial seeds before UV irradiation (green) and artificial seeds after the UV irradiation (blue).

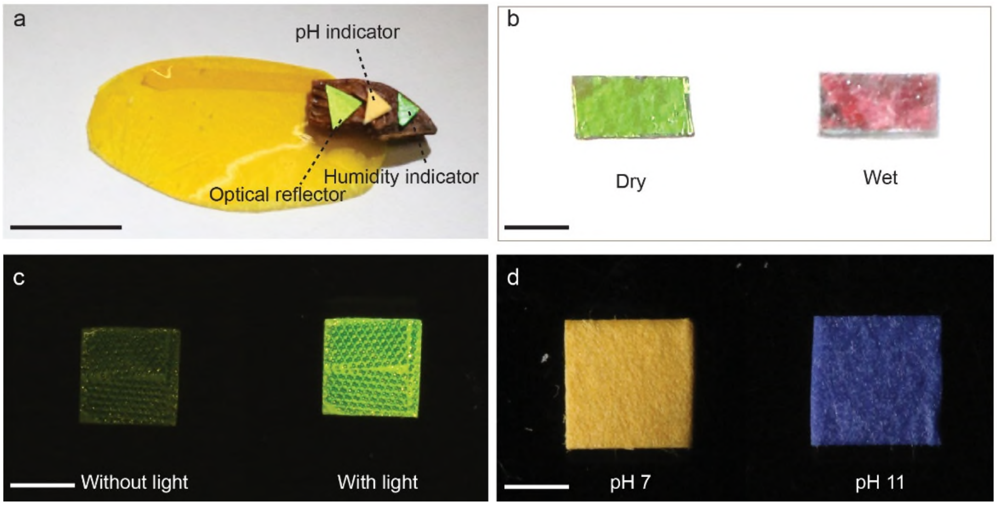

Supplementary Fig. 17 | Wind-dispersed indicators integration. (**a**) The picture of an artificial seed with three kinds of indicators integrated on board. Scale bar is 1 cm. (**b**) The color change of a humidity responsive CLC film under different humidity conditions. (**c**) Comparison of brightness of an optical tracker in dark and upon light illumination. (**d**) Color change of color of a pH indicator at different pH values. All scale bars in (**b**, **c**, **d**) are 2.5 mm.

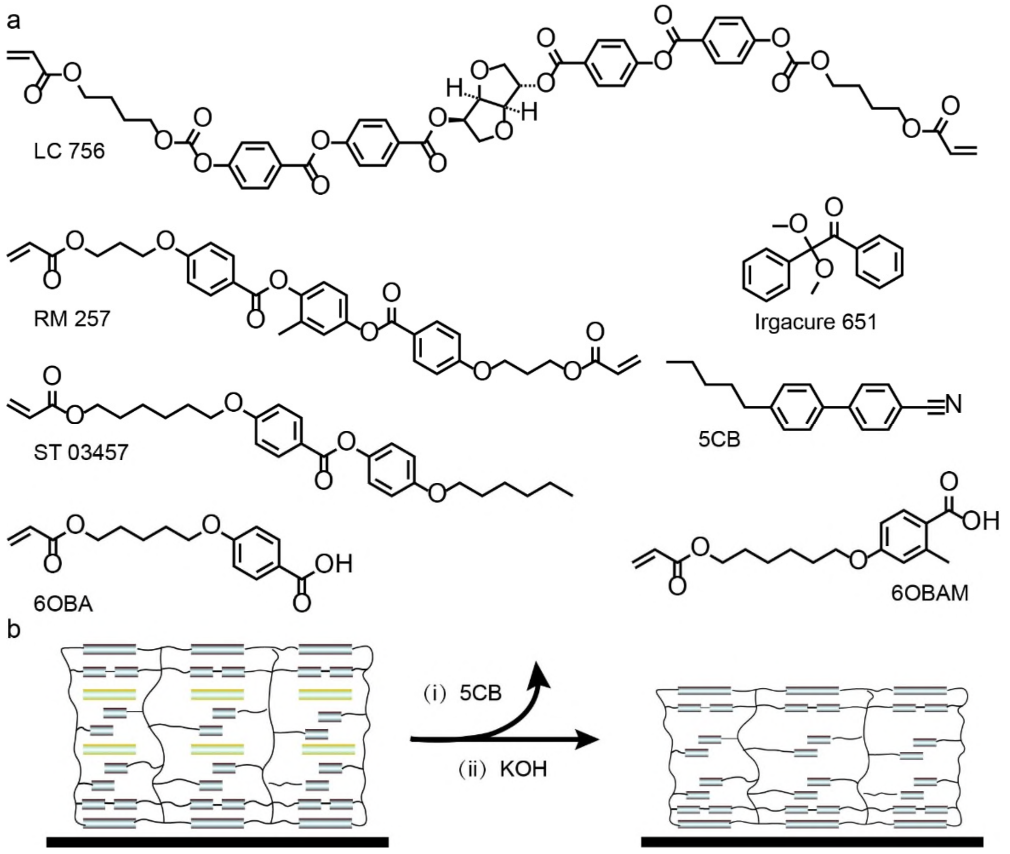

Supplementary Fig. 18 | Synthetic steps of humidity-responsive cholesteric liquid-crystalline (CLC). (**a**) Chemical structure of all molecules in use. Pre-cured mixture contains 17.9 wt% RM 257, 22.9 wt% ST 03457, 18 wt% of each of 6OBA, 6OBAM and 5CB, 4.6 wt% LC 756 and 0.6 wt% photoinitiator. (**b**) Schematic drawing of the humidity-responsive CLC preparation process.

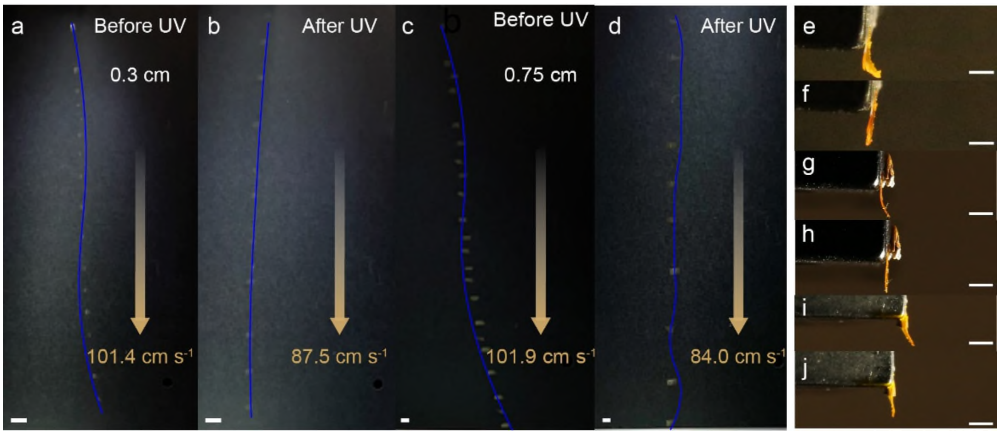

Supplementary Fig. 19 | Light tuning in artificial seeds of different sizes. Superimposed images of a free-falling artificial seed (size: 0.3 cm) before (**a**) and after (**b**) UV irradiation. Superimposed images of a free-falling artificial seed (size: 0.75 cm) before (**c**) and after (**d**) UV irradiation. All scale bars in (**a-d**) are 0.5 mm. The shape of the artificial seed wing (size: 0.3 cm) before (**e**) and after (**f**) UV irradiation. The shape of the artificial seed wing (size: 0.75 cm) before (**g**) and after (**h**) UV irradiation. The shape of the artificial seed wing (size: 1.5 cm) before (**i**) and after (**j**) UV irradiation. All scale bars in (**e-j**) are 0.25 mm. UV: 240 mW cm^{-2}, 20 s illumination.

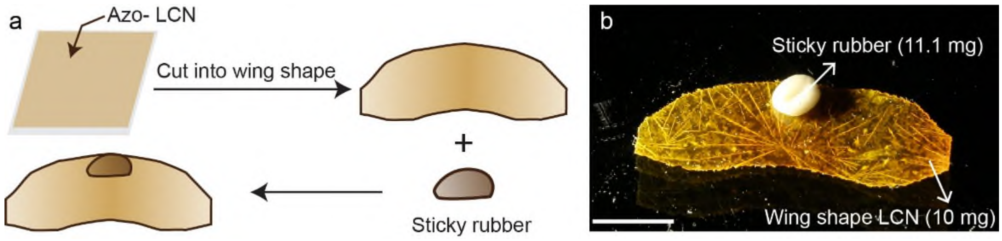

Supplementary Fig. 20 | Fabrication of artificial Javan cucumber seed. (**a**) The Schematic drawing of steps of fabrication of artificial Javan cucumber seed. (**b**) The picture of artificial Javan cucumber seed. The scale bar is 5 mm.

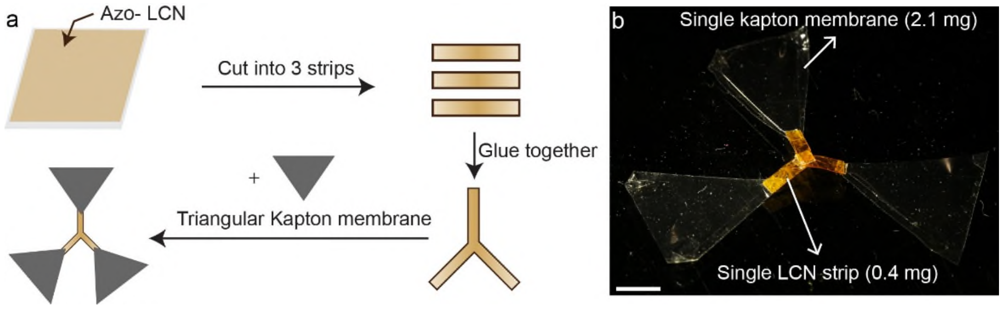

Supplementary Fig. 21 | Fabrication of LCN-membrane parachute. (**a**) The Schematic drawing of steps of fabrication of LCN-membrane parachute. (**b**) The picture of LCN-membrane parachute. The scale bar is 5 mm.

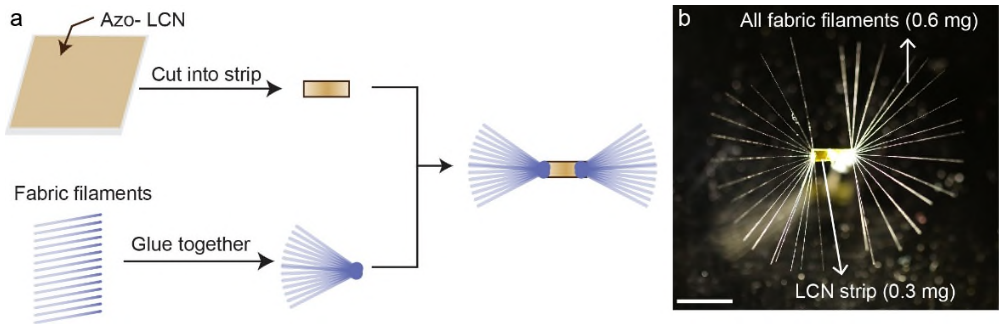

Supplementary Fig. 22 | Fabrication of artificial dandelion seed. (**a**) The Schematic drawing of steps of fabrication of artificial dandelion seed. (**b**) The picture of artificial dandelion seed. The scale bar is 5 mm.

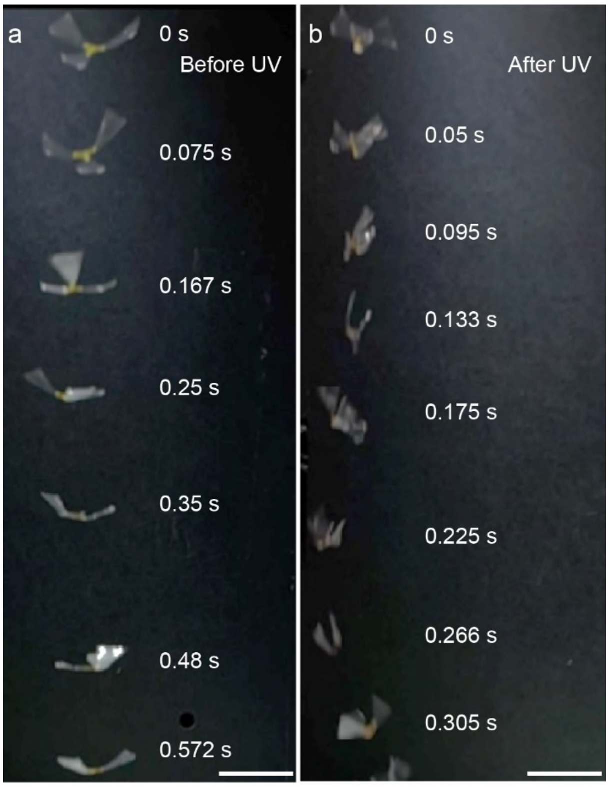

Supplementary Fig. 23 | The free-falling experiment of LCN-membrane parachute. Superimposed images of a free-falling of LCN-membrane parachute before (**a**) and after (**b**) UV illumination (150 mW cm^{-2}, 20 s). All scale bars are 4 cm.

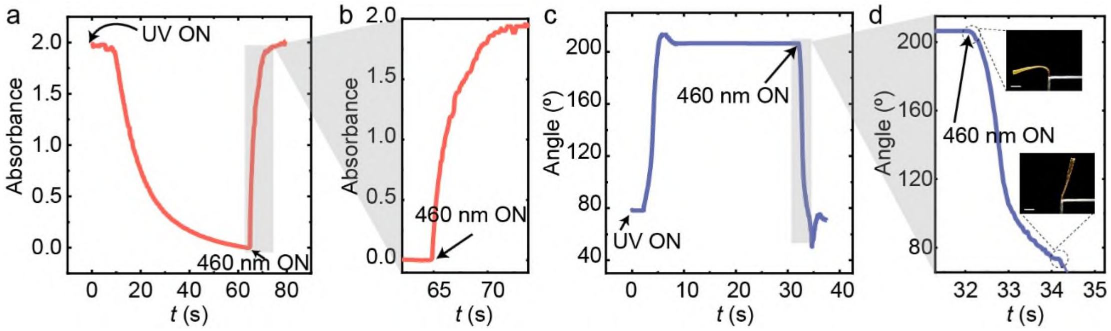

Supplementary Fig. 24 | Photo-response of azo-LCN. (**a**) UV–Vis spectra of a solid actuator film upon UV (150 mW cm^{-2}) and 460 nm (300 mW cm^{-2}) illumination. Film thickness: 5 µm. (**b**) Spectral details at the moment upon switching on 460 nm illumination. (**c**) Deformation kinetics of the azo-LCN strip under UV (150 mW cm^{-2}) and 460 nm (300 mW cm^{-2}) illumination. Strip thickness: 20 µm. (**d**) The zoomed-in view of angle change of azo-LCN strip upon switching on 460 nm illumination. Insets: photographs of the actuator strip at the deformed and relaxed stages. The scale bars are 5 mm.

2. Supplementary Table

Model	ExpGro1
Equation	y=A1*exp(x/t1)+y0
Plot	B
y0	1.85246±4.629E-4
A1	-0.97813±7.44096E-4
t1	-303.4554±0.58882
Reducedd Chi-Sqr	7.91E-4
R-Square(COD)	0.98725
Adj.RSquare	0.98725

Supplementary Table 1 | The cis-lifetime of LCN film is determined by using an exponential fit with decay time about 303 min, the thickness of LCN flim: 5 µm.

	Weight (mg)	Terminal velocity (cm s^{-1})	Actuation mechanism	Control in air	Continuous shape-morphing	Response time
Origami microfliers[2]	414	140~ 180	Solar electricity and electromagnetism	Yes	No	25 ms
Artificial dandelion[3]	4	41.0~ 98.0	Light-heat induced gas desorption	Yes	Yes	2.5 s
Rotary flight[4]	5.3	72.9~ 80.0	Light-heat induced gas desorption	No	No	650 ms
**Fliers in this study**						
Artificial maple samara	32.1	86.8~ 98.4	Photochemical effect	Yes	Yes	< 3 s
Artificial glider	24.8	60.0~ 88.3	Photochemical effect	No	Yes	< 3 s
Parachute	9.2	70.7~ 115.4	Photochemical effect	No	Yes	< 3 s
Artificial dandelion seed	1.2	45.8~ 81.2	Photochemical effect	Yes	Yes	< 3 s

Supplementary Table 2 | Comparison of microfliers based on passive flight mode.

3. Supplementary Methods

Fabrication of cholesteric liquid crystalline film

The cholesteric liquid crystalline mixture contains 17.9 wt% RM 257, 22.9 wt% ST 03457, 18 wt% of each of 6OBA, 6OBAM and 5CB, 4.6 wt% LC 756 and 0.6 wt% photoinitiator. The mixture was dissolved in tetrahydrofuran solution, then dropped on PVA-glass. After evaporating the solvent at 75°C, another piece of glass was placed on top and cooled to room temperature. Shearing the upper glass enables orientation of LC molecules into a cholesteric nematic phase. Photopolymerization was done by irradiating the sample under UV light (365 nm, 50 mW cm^{-2}) for 5 min. Then, the upper glass was removed. 5CB was first evaporated at 120 °C and then treated with 1 M KOH to obtain a humidity responsive cholesteric liquid crystal film.

Fabrication of artificial Javan cucumber seed

The azo-LCN film was trimmed into the form of a Javan cucumber seed wing. Then, a sticky rubber was placed in the centre of it to serve as the artificial seed. The width of the artificial Javan cucumber seed was 2 cm, thickness is 0.02 mm.

Fabrication of LCN-membrane parachute

Three azo-LCN strips were glued together. Triangular Kapton wings were glued to the end of each strip. Each azo-LCN film dimensions: 0.5 cm × 0.15 cm × 0.02 mm.

Fabrication of artificial dandelion seed

Fabric filaments were UV-glued together to form a semicircle. Two bristle semicircles were subsequently affixed to both ends of an azo-LCN film to construct the artificial dandelion seed. Azo-LCN film dimension: 0.35 cm × 0.15 cm × 0.02 mm.

4. Supplementary references

1. Sohn, M.H., Im, D.K. Flight characteristics and flow structure of the autorotating maple seeds. *J Vis* **25**, 483–500 (2022).
2. Johnson, K., *et al.* Solar-powered shape-changing origami microfliers. *Sci. Robot.* **8**, eadg4276 (2023).
3. Chen, Y., Valenzuela, C., Zhang, X., Yang, X., Wang, L., Feng, W. Light-driven dandelion-inspired microfliers. *Nat. Commun.* **14**, 3036 (2023).
4. Wang, D., *et al.* Bioinspired rotary flight of light-driven composite films. *Nat. Commun.* **14**, 5070 (2023).
