# Supplementary material for: Photochemically responsive polymer films enable tunable gliding flights
Source: Nat Commun. 2024 Jun 1;15:4684. doi: 10.1038/s41467-024-49108-0 (PMC11144244; doi:10.1038/s41467-024-49108-0)
Supplement: Supplementary file 3 — Description of Additional Supplementary Files [file 41467_2024_49108_MOESM3_ESM.pdf]

Description of Additional Supplementary Files

File Name: Supplementary Movie 1
Description: Free-fall experiment of natural and artificial maple samara.

File Name: Supplementary Movie 2
Description: Actuation kinetics of azo-LCN strip under UV illumination.

File Name: Supplementary Movie 3
Description: Free-fall experiment of an artificial maple samara before and after UV illumination.

File Name: Supplementary Movie 4
Description: The reversible tunning of autorotation inside a wind tunnel.
